# Development of a split fluorescent protein-based RNA live-cell imaging system to visualize mRNA distribution in plants

**DOI:** 10.1186/s13007-022-00849-3

**Published:** 2022-02-08

**Authors:** Nien-Chen Huang, Kai-Ren Luo, Tien-Shin Yu

**Affiliations:** Institute of Plant and Microbial Biology, Academia Sinica, Taipei, 11529 Taiwan

**Keywords:** RNA live-cell imaging, RNA-binding protein, Split fluorescent proteins, Mobile mRNAs, Plasmodesmata, Agro-infiltration

## Abstract

**Background:**

RNA live-cell imaging systems have been used to visualize subcellular mRNA distribution in living cells. The RNA-binding protein (RBP)-based RNA imaging system exploits specific RBP and the corresponding RNA recognition sequences to indirectly label mRNAs. Co-expression of fluorescent protein-fused RBP and target mRNA conjugated with corresponding RNA recognition sequences allows for visualizing mRNAs by confocal microscopy. To minimize the background fluorescence in the cytosol, the nuclear localization sequence has been used to sequester the RBP not bound to mRNA in the nucleus. However, strong fluorescence in the nucleus may limit the visualization of nucleus-localized RNA and sometimes may interfere in detecting fluorescence signals in the cytosol, especially in cells with low signal-to-noise ratio.

**Results:**

We eliminated the background fluorescence in the nucleus by using the split fluorescent protein-based approach. We fused two different RBPs with the N- or C-terminus of split fluorescent proteins (FPs). Co-expression of RBPs with the target mRNA conjugated with the corresponding RNA recognition sequences can bring split FPs together to reconstitute functional FPs for visualizing target mRNAs. We optimized the system with minimal background fluorescence and used the imaging system to visualize mRNAs in living plant cells.

**Conclusions:**

We established a background-free RNA live-cell imaging system that provides a platform to visualize subcellular mRNA distribution in living plant cells.

## Background

Most cells transport mRNAs to specific subcellular compartments for localized RNA translation. Instead of transporting a quantity of proteins, the transport of mRNA provides an efficient strategy to distribute proteins to specific subcellular compartments. In eukaryotic cells, asymmetric mRNA localization is a conserved mechanism to create translation hotspots for regulating spatial gene expression in response to environmental cues [1]. The intracellular mRNA asymmetric distribution in plants can be extended to the intercellular level in which specific plant mobile mRNAs are transcribed in local tissues and undergo long-distance movement via phloem to exert non-cell autonomous functions, including regulation of leaf development [2], floral transition [3, 4], and potato tuber formation [5]. Thus, understanding the mRNA subcellular distribution may provide information to unravel RNA logistics and post-transcriptional gene regulation.

In living plant cells, direct visualizing of mRNAs by microscopy is challenging. Many approaches have been developed that allow visualizing mRNAs in living cells. Direct labeling of mRNAs can be achieved by incorporating in vitro-synthesized mRNA with fluorescein-labeled nucleotides or introducing molecular beacons into cells to hybridize with the target mRNA [6, 7]. However, these in vitro RNA labeling systems require invasive injection of labeled RNA into plant cells such as microinjection or particle bombardment. In addition, the in vitro-synthesized RNAs may behave differently from the in vivo-transcribed RNAs because the RNA synthesized in vitro may bypass the RNA modification occurring in the nucleus, which may change the properties of RNA.

Alternatively, RNA can be indirectly visualized by an RNA aptamer-based approach. In this system, the target mRNA is genetically modified by tagging with an RNA aptamer recognized by RNA-binding proteins (RBPs). Co-expression of fluorescent proteins (FPs) conjugated with RBPs with aptamer-tagged mRNA allows for indirect labelling of mRNA in living cells. MCP, a coat protein derived from MS2 bacteriophage with RNA-binding activity, and the stem loop structures recognized by MCP have been widely used to monitor mRNA localization in yeast, fruit fly, mammals and plants [8–13]. To reduce the background GFP fluorescence detected in the cytosol, the nuclear localization sequence (NLS) has been used to sequester the MCP-GFP not bound to RNA in the nucleus [8, 13]. In addition, the signal-to-noise ratio of RNA detected in the cytosol can be greatly improved by the plant-specific NLS tightly restricting MCP in the nucleus [13].

**Fig. 1:**
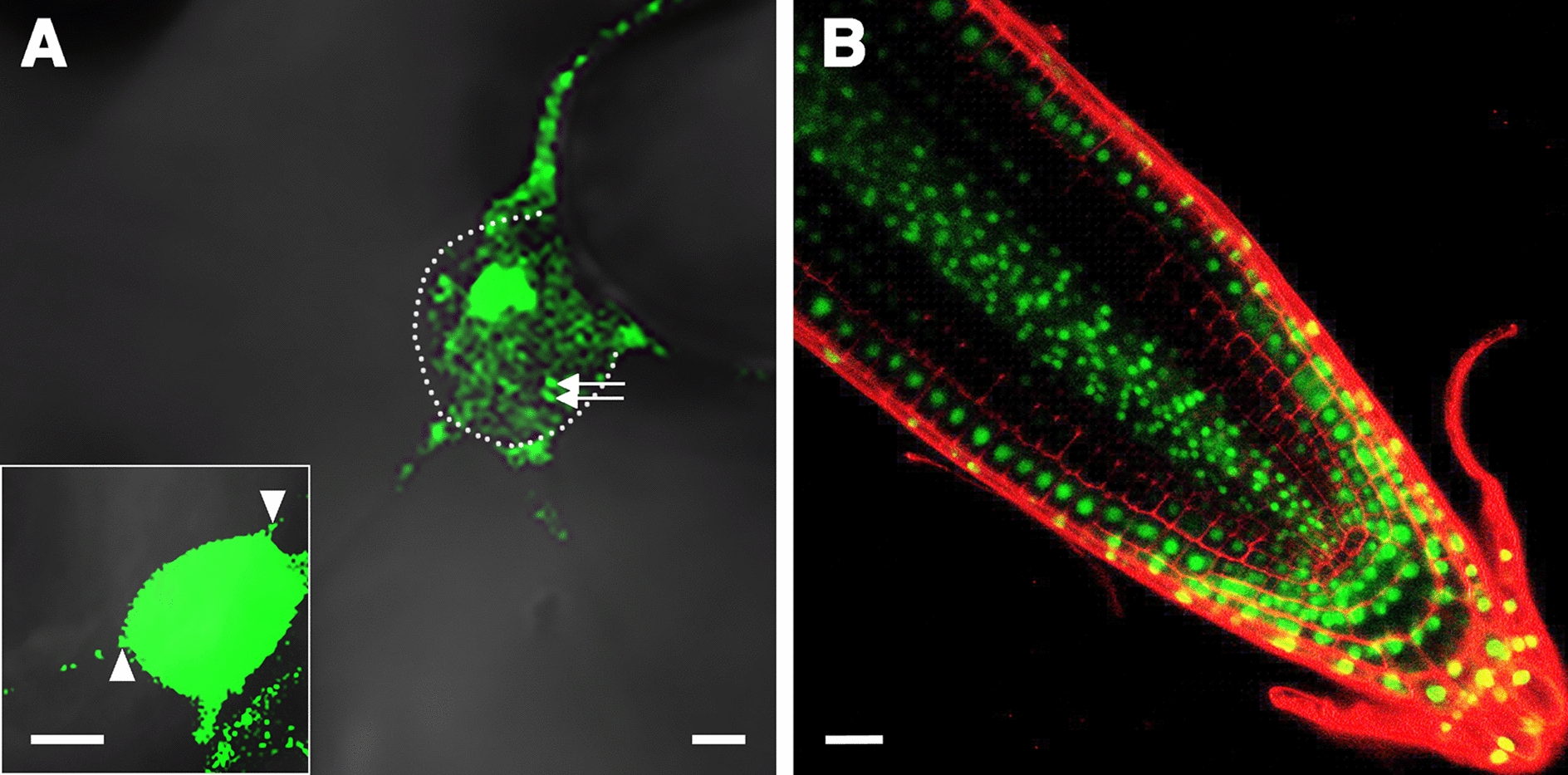
Background GFP fluorescence disturbs observation of labeled mRNA. **A** Confocal microscopy of *N. benthamiana* leaves co-infiltrated with nucleus-localized MCP_FD_-GFP and FT-SL24, which is the coding sequences of Arabidopsis *FLOWERING LOCUS T* (*FT*) conjugated with 24 repeats of stem loop (SL) structures recognized by MCP [13]. The background fluorescence derived from nucleus-localized MCP-GFP interferes with the detection of potential *FT* mRNA foci (arrows) in the nucleus. Nucleus boundary is outlined with white dots. Bar = 2 μm. Inset, in cells with high accumulation level of MCP-GFP in the nucleus, high nucleus-to-cytoplasmic ratio of GFP fluorescence disturbs detection of *FT* mRNA foci (arrowheads) proximal to nucleus. Bar = 5 μm. **B** Roots of Arabidopsis transformant harboring constitutively expressed MCP_FD_-GFP. Note that in cells with high nucleus-to-cytoplasmic ratio, the background GFP fluorescence may interfere in mRNA localization in the cytosol. Scale bar = 20 mm

Although the MCP-based approach has been successfully used to visualize the intracellular distribution of mobile and non-mobile mRNA in plants [13], the background GFP fluorescence derived from nucleus-localized MCP-GFP may interfere with detecting mRNA located close to the nucleus (Fig. 1A). In addition, cells that undergo active cell division are smaller and the nucleus is relatively large [14], so the high nucleus-to-cytoplasmic ratio may disturb the detection of RNA localization due to strong background GFP fluorescence in the nucleus (Fig. 1B).

In this study, we improved the RNA live-cell imaging system by eliminating the background GFP fluorescent in the nucleus. The application of this split FPs-based system detected differential subcellular distribution between mobile and non-mobile mRNAs. In addition, the split FPs-based system greatly improved the signal-to-noise ratio in the nucleus, which confers detection of mRNAs in the nucleus or at juxtanuclear region. Thus, our system broadens the application of RNA live-cell imaging system in plants.

## Results and discussion

### Development of split FP-based RNA live-cell imaging system

**Fig. 2:**
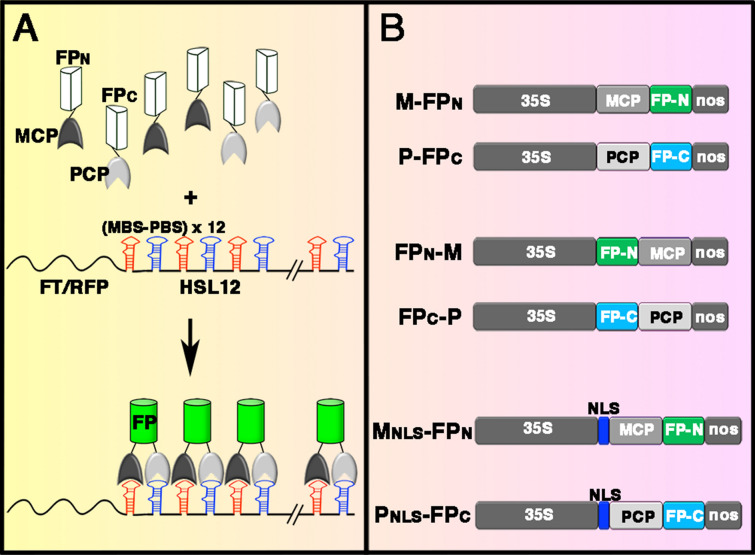
Illustration of split fluorescent protein (FP)-based RNA live-cell imaging system. **A** MCP or PCP, the coat proteins of MS2 or PP7 bacteriophage, was fused with the N- or C-terminus of split FPs, FP_N_ or FP_C_, respectively. The *FT* or *RFP* mRNA was conjugated with 12 copies of MBS-PBS, the hairpin structure recognized by MCP and PCP, to generate *FT*_*HSL12*_ or *RFP*_*HSL12*_ chimeric RNA. The binding of MCP and PCP with the hairpin structure of MBS-PBS brings two split FPs together to reconstitute the functional FP for indirect visualization of mRNAs in living cells. **B** Illustration of the constructs of MCP (M) and PCP (P) fused with the N- or C-terminus of FP_N_ or FP_C_. The nuclear localization sequence (NLS) from Arabidopsis FD was inserted to sequester MCP- or PCP-FP fusion proteins in the nucleus. The constructs were driven by a CaMV35S promoter and had an NOS terminator

To develop the mRNA live-cell imaging system without background GFP fluorescence in the nucleus, we exploited the bimolecular fluorescence complementation system to reduce background GFP fluorescence derived from MCP-GFP not bound to mRNA. In this system, two different RBPs were fused with the N- or C-terminus of split FPs to reduce the fluorescence produced from RBP-FPs not bound to mRNA. The target mRNA was flanked with the corresponding RNA recognition sequences of the RBPs. The interaction between RBPs and RNA recognition sequences may bring two split FPs together to reconstitute a mature FP (Fig. 2A).

We selected two coat proteins (CPs) of MS2 and PP7 bacteriophages, MCP and PCP, which specifically bind to an RNA hairpin structure of MBS and PBS, respectively [15]. MCP and PCP were conjugated with the N-terminus fragment (FP_N_, amino acids 1–173 from Venus) or C-terminus fragment (FP_C_, amino acids 156–239 from SCFP3A) of the split FP protein [16]. To identify the optimized fusion proteins for the system, we generated different MCP/PCP-FP fusion proteins by flanking FP_N_ or FP_C_ with MCP or PCP (Fig. 2B). In addition, the NLS derived from Arabidopsis FD [13], which encodes a bZIP transcription factor [17], was used to locate MCP or PCP to the nucleus (Fig. 2B). To generate the target mRNAs that can be recognized by MCP and PCP, the DNA fragments containing 12 copies of MBS-PBS tandem repeats were conjugated with *FT* or *RFP* cDNA to create *FT*_*HSL12*_ (*FT*-hybrid stem loops, 12 copies) or *RFP*_*HSL12*_, respectively (Fig. 2A).

### Background GFP fluorescence was under the detection limit in cells expressing MCP-FP_N_ and PCP-FP_C_

**Fig. 3:**
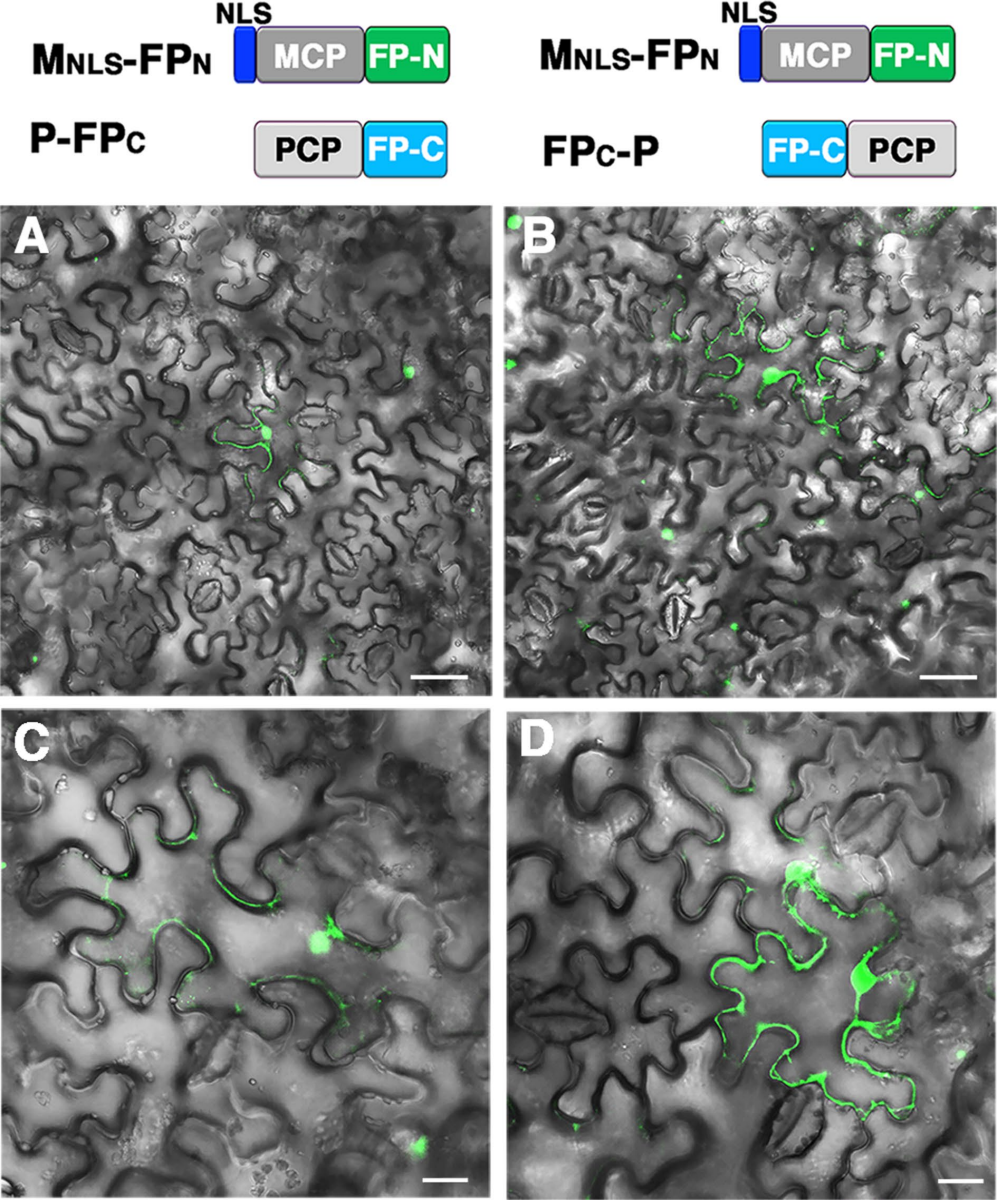
Detection of background GFP fluorescence in cells with co-expression of PCP-FP and NLS containing MCP-FP. Confocal microscopy of *N. benthamiana* leaves co-infiltrated with **A**, **C** M_NLS_-FP_N_ and P-FP_C_ or **B**, **D** M_NLS_-FP_N_ and FP_C_-P. **C** and **D** Magnified images of **A** and **B**, respectively. Note that background GFP fluorescent is detected in both the cytosol and nucleus. Scale bar: **A** and **B** = 50 mm; **C** and **D** = 20 mm

**Fig. 4:**
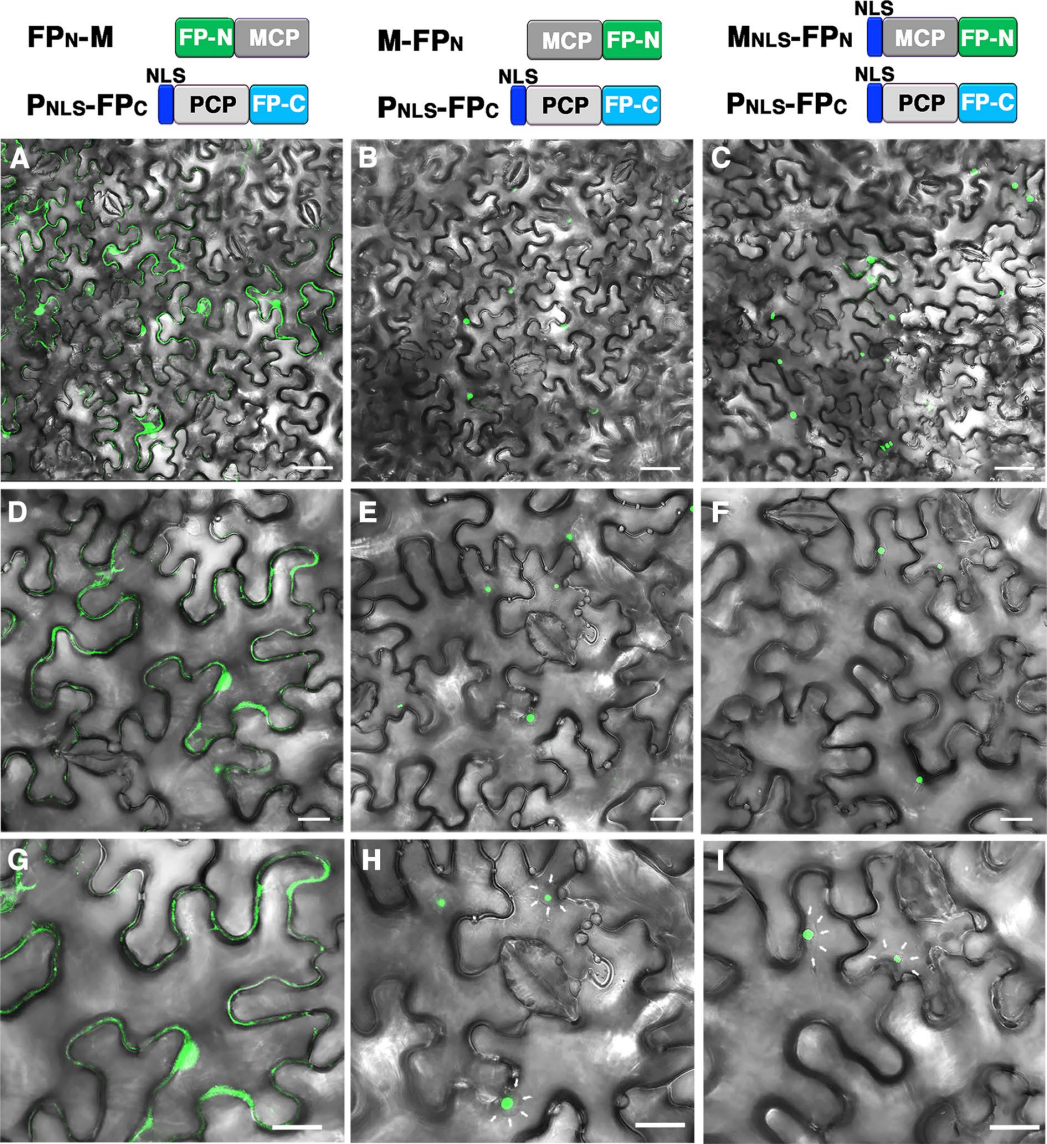
Speckle-like background GFP fluorescence in nucleus of cells with co-expression of MCP-FP and NLS-containing PCP-FP. Confocal microscopy of *N. benthamiana* leaves co-infiltrated with **A**, **D**, **G** FP_N_-M and P_NLS_-FP_C_; **B**, **E**, **H** M-FP_N_ and P_NLS_-FP_C_; or **C**, **F**, **I** M_NLS_-FP_N_ and P_NLS_-FP_C_. **D**–**I** Magnified images of **A–C**. Note that the background GFP fluorescence is detected in both the cytosol and nucleus in **A**, **D**, **G**, whereas the speckle-like background GFP fluorescence is detected in the nucleus in **B**, **C**, **E**, **F**, **H**, **I**. Scale bar: **A**–**C** = 50 mm; **D**–**I** = 20 mm. The boundary of the nucleus is indicated by white arrows in **H** and **I**

To identify the combinations of MCP and PCP with minimal background GFP fluorescence in plant cells, we transiently expressed different MCP- and PCP-split FPs in leaves of 3-weak-old *Nicotiana benthamiana* by agro-infiltration and examined GFP fluorescence under a confocal laser scanning microscope at 3 days after infiltration. When NLS-containing M_NLS_-FP_N_ was co-expressed with P-FP_C_ or FP_C_-P, weak GFP fluorescence was detected in both the cytosol and nucleus (Fig. 3A–D), which suggests that the separation of MCP and PCP into different subcellular compartments may not significantly reduce the background GFP fluorescence. Consistently, when NLS-containing P_NLS_-FP_C_ was co-expressed with FP_N_-M, the background GFP fluorescence was again detected in the cytosol and nucleus (Fig. 4A, D, G). However, when P_NLS_-FP_C_ was co-expressed with M-FP_N_ or NLS-containing M_NLS_-FP_N_, the background GFP fluorescence that formed a speckle-like spot was detected only in the nucleus (Fig. 4B, C, E, F, H, I), which suggests that the topology of fusion proteins rather than subcellular localization may contribute to the background GFP fluorescence.

**Fig. 5:**
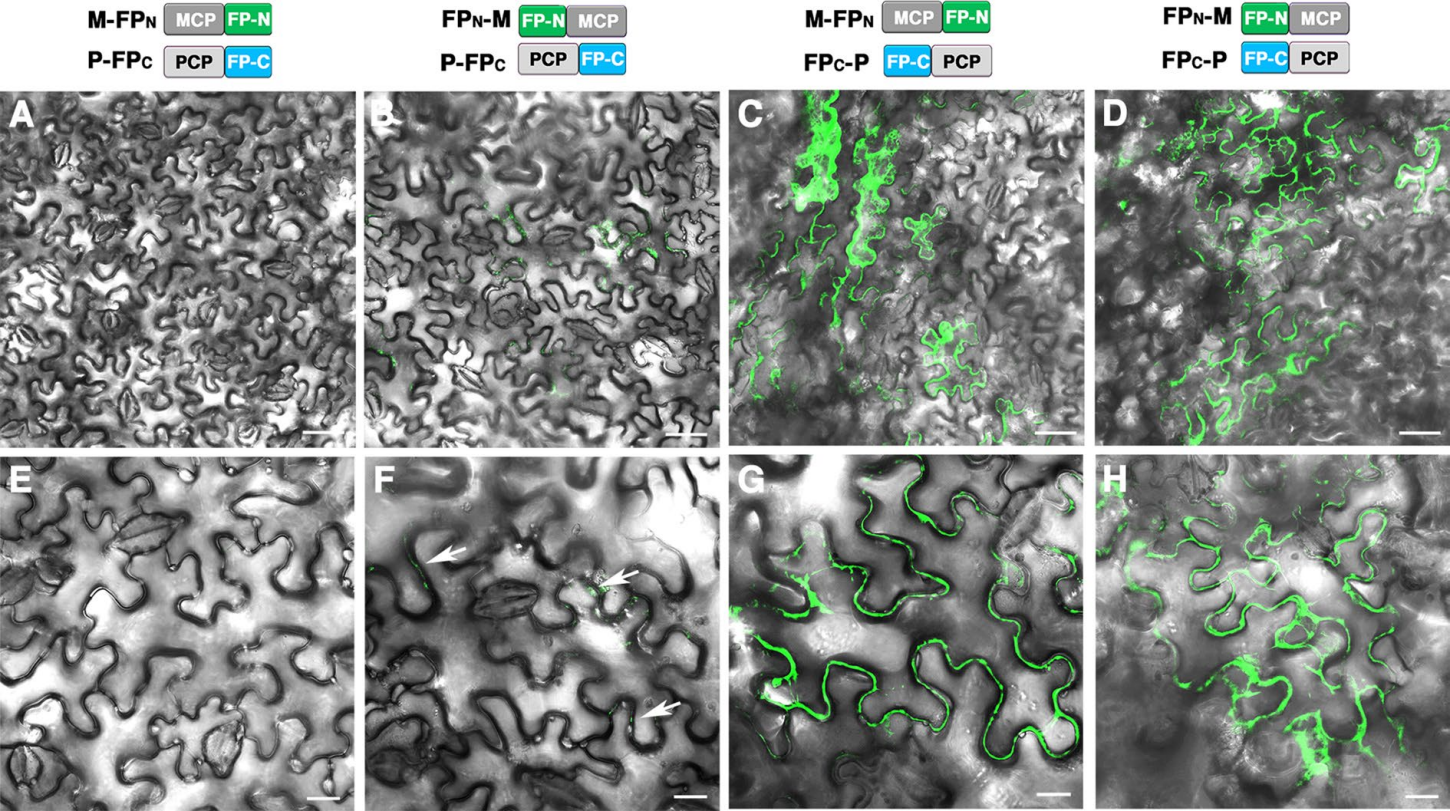
GFP background fluorescence was not detected in cells with co-expression of M-FP_N_ and P-FP_C._ Confocal microscopy of *N. benthamiana* leaves co-infiltrated with **A**, **E** M-FP_N_ and P-FP_C_; **B**, **F** FP_N_-M and P-FP_C_; **C**, **G** M-FP_N_ and FP_C_-P; or **D**, **H** FP_N_-M and FP_C_-P. **E**–**H** Magnified images of **A**–**D**. Note that background GFP is under the detection limit in **A**, **E**. The weak background GFP fluorescence is indicated by white arrows in **F**. Scale bar: **A**–**D** = 50 mm; **E**–H = 20 mm

We next examined background GFP fluorescence in cells expressing MCP-FP and PCP-FP without NLS. When M-FP_N_ was co-expressed with P-FP_C_, we consistently obtained the images without detectable background GFP fluorescence (Fig. 5A and E). However, the other 3 combinations of MCP-FP and PCP-FP conferred weak (Fig. 5B and F) or strong GFP fluorescence (Fig. 5C, D, and G, H). We summarize these results in Table 1. Taken together, our results reveal that the combination of MCP-FP_N_ and PCP-FP_C_ produced undetectable background GFP fluorescence. In addition, the combination of P_NLS_-FP_C_ with M-FP_N_ or M_NLS_-FP_N_ produced speckle-like background GFP fluorescence in the nucleus, which may also provide a useful positive control for the presence of the infiltrated constructs.

**Table 1:** Summary of background GFP fluorescence detection with different combinations of split fluorescent proteins

MCP varieties	PCP varieties	Background	Fluorescence
M_NLS_-FP_N_	P-FP_C_	Yes	Nucleus, cytosol
M_NLS_-FP_N_	FP_C_-P	Yes	Nucleus, cytosol
FP_N_-M	P_NLS_-FP_C_	Yes	Nucleus, cytosol
M-FP_N_	P_NLS_-FP_C_	Yes	Speckle in nucleus
M_NLS_-FP_N_	P_NLS_-FP_C_	Yes	Speckle in nucleus
M-FP_N_	P-FP_C_	No	Non-detectable
FP_N_-M	P-FP_C_	Yes	Cytosol
M-FP_N_	FP_C_-P	Yes	Nucleus, cytosol
FP_N_-M	FP_C_-P	Yes	Cytosol

### Detection of mRNA localization in background-free split FP-based imaging system

**Fig. 6:**
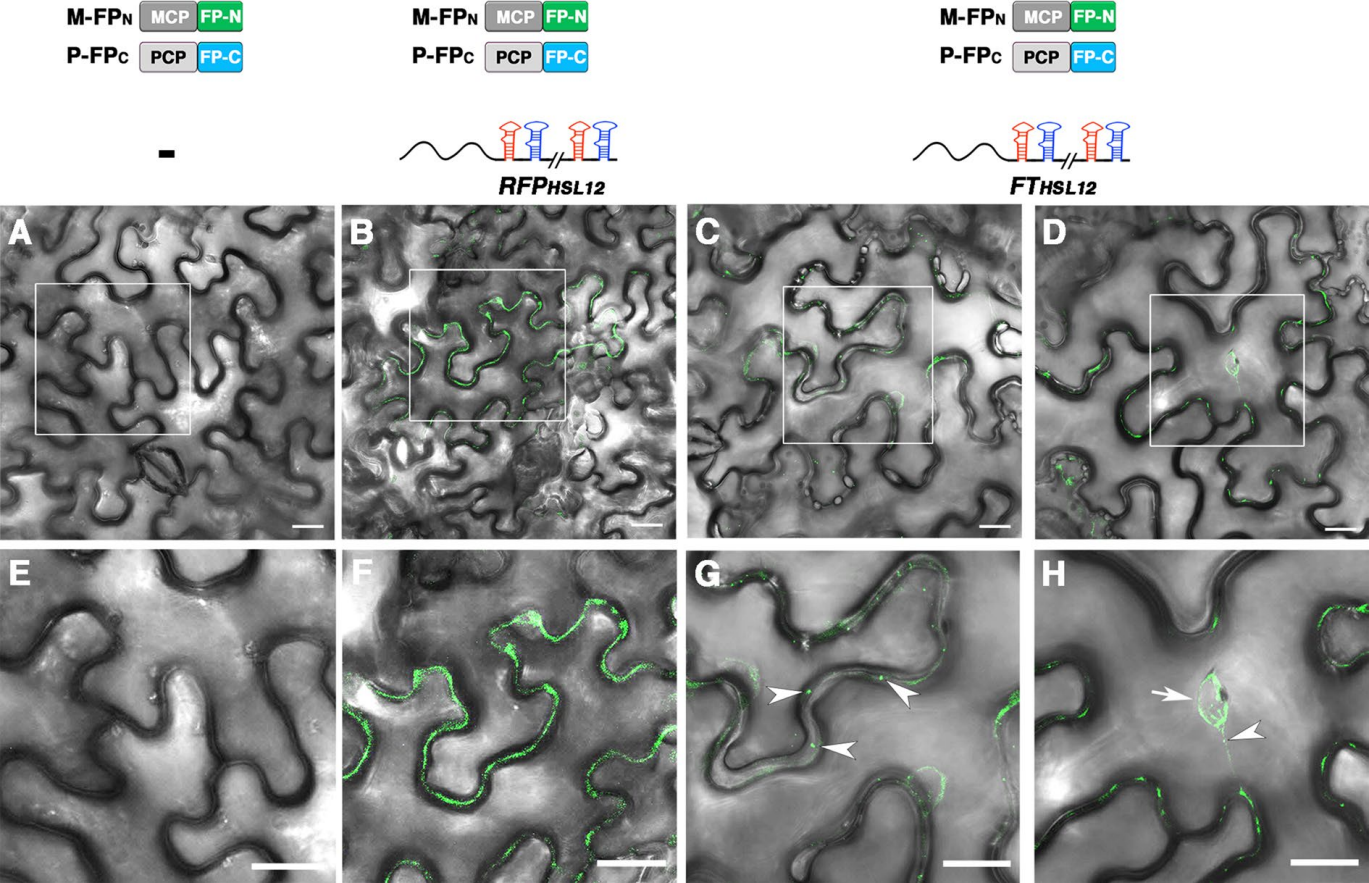
Visualization of mRNA distribution by split FP-based RNA live-cell imaging system. Confocal microscopy of *N. benthamiana* leaves co-expressing **A**, **E** M-FP_N_ and P-FP_C_ without target mRNA; **B**, **F** M-FP_N_ and P-FP_C_ with *RFP*_*HSL12*_; or **C**, **D**, **G, H** M-FP_N_ and P-FP_C_ with *FT*_*HSL12*_ RNA. **E**–**H** are the magnified images of the rectangle marked area in **A**–**D**. Different fluorescence distribution was detected in cells expressing *RFP*_*HSL12*_ or *FT*_*HSL12*_ RNA. **G** The punctate GFP fluorescence in the cell periphery is indicated by arrowheads. **H** The GFP fluorescence spot located on nucleus boundary or transported along cytoplasmic strands is indicated by an arrow or arrowhead, respectively. Scale bar = 20 mm in all images

To verify the application of the split-FP system for localization of mRNAs, we co-infiltrated M-FP_N_ and P-FP_C_ with *FT*_*HSL12*_ or *RFP *_*HSL12*_, two mRNAs with differential subcellular distribution pattern [13] in tobacco leaves. At 3 days after infiltration, no fluorescence was detected in control cells co-expressing M-FP_N_ and P-FP_C_ without mRNA (Fig. 6A, E). However, GFP fluorescence was detected in the cytosol of cells co-expressing M-FP_N_ and P-FP_C_ with *RFP *_*HSL12*_ or *FT*_*HSL12*_ mRNA. Of note, the fluorescence in the cytosol showed distinct distribution patterns in cells expressing *RFP *_*HSL12*_ or *FT*_*HSL12*_ mRNA: in cells expressing *RFP *_*HSL12*_, the fluorescence was evenly distributed at the cell periphery (Fig. 6B, F), whereas with *FT*_*HSL12*_, the fluorescence showed a punctate distribution (Fig. 6C, G). These subcellular distribution patterns were reminiscent of previous MS2-based mRNA live-cell imaging [13]. However, in the split FP-based imaging system, the GFP fluorescence spots were detected inside or in the boundary of the nucleus (Fig. 6D–H), which were not detected in the previous MS2-based system [13]. These results suggest the broader use of a split FP-based imaging system for visualizing mRNA distribution.

**Fig. 7:**
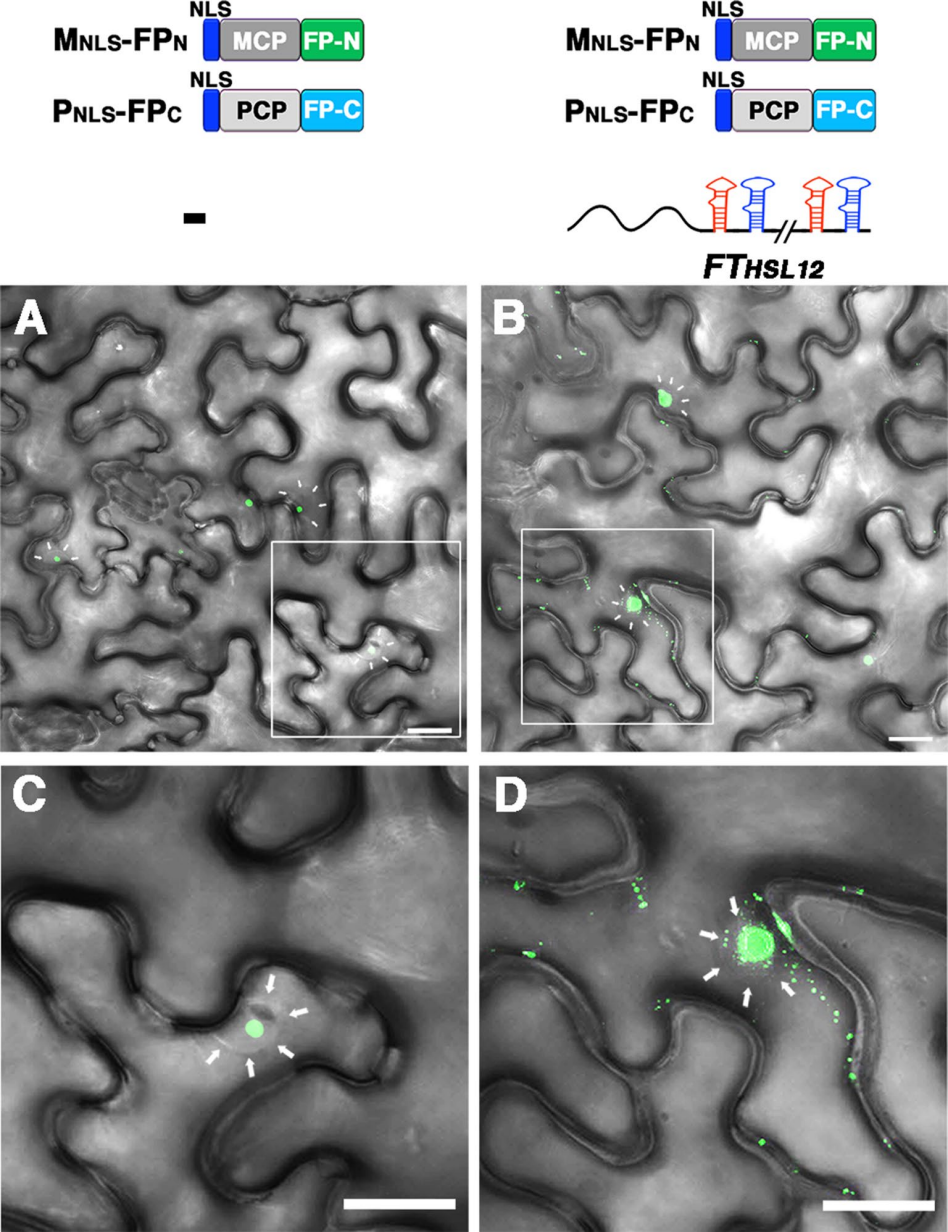
Visualization of mRNA distribution in cells co-expressing M_NLS_-FP_N_ and P_NLS_-FP_C_ with *FT*_*HSL12*_ mRNA. Confocal microscopy of *N. benthamiana* leaves co-expressing **A**, **C** M_NLS_-FP_N_ and P_NLS_-FP_C_ without target mRNA or **B**, **D** with *FT*_*HSL12*_. **C** and **D** are the magnified images of the rectangle marked area in **A** and **B**, respectively. Note that the punctate fluorescence of *FT*_*HSL12*_ mRNA is detected in the nucleus and along cytoplasmic strands or cell periphery in **D**. The boundary of the nucleus is indicated by arrows in **C** and **D**. Scale Bar = 20 mm in all images

We further verified the application of the split FP-based imaging system with speckle-like background GFP fluorescence for mRNA visualization. In control cells co-expressing M_NLS_-FP_N_ and P_NLS_-FP_C_, speckle-like GFP fluorescence was detected in the nucleus (Fig. 7A and C), whereas in cells co-expressing M_NLS_-FP_N_ and P_NLS_-FP_C_ with *FT*_*HSL12*_ mRNA, the GFP fluorescence was detected in the cytosol with a punctate distribution pattern (Fig. 7B and D). Of note, GFP fluorescence spots were detected in the nucleus (Fig. 7D), suggesting that the speckle-like background GFP may not greatly disturb mRNA visualization in the nucleus.

## Conclusions

We have established a split FP-based RNA live-cell imaging system for visualizing subcellular mRNA distribution in living plant cells. This system eliminates the background GFP fluorescence in the nucleus. We applied this system to monitor intracellular transport of mobile and non-mobile mRNA. In addition, this system enables the detection of mRNAs near the nucleus. Our method broadens the application of RBP-based RNA imaging system in living plant cells.

## Methods

### Plant materials and growth conditions

*Nicotiana benthamiana* plants were grown in a growth chamber under 27 °C, 16-h/8-h day/night cycles and light intensity 100 μmol m^−2^ s^−1^. The 3-week-old plants were used for agro-infiltration assay.

### Plasmid construction and Arabidopsis transformation

The DNA fragments of MCP and PCP were PCR-amplified with the following primers: MCP-For (5’-GGATCCATGGCTTCTAACTTTACTCAGTTCG-3’), MCP-Rev (5’-CTCGAGGTAGATGCCGGAGTTTGCTGCGATT-3’), PCP-For (5’-GGATCCATGTCCAAAACCATCGTTCTTTCGG-3’), PCP-Rev (5’-CTCGAGACGGCCCAGCGGCACAAGGTTGACG-3’). The DNA fragments of MCP and PCP were confirmed by sequencing and cloned into split FP-containing binary vectors pVYNE, pVYNE(R), pSCYCE, or pSCYCE(R) [16] to generate M-FP_N_, FP_N_-M, P-FP_C_ and FP_C_-P, respectively. The C-terminal fragment (211–240 a.a.) of Arabidopsis FD, which contains the nuclear localization sequence (NLS) [13], was inserted in M-FP_N_ or P-FP_C_ to generate M_NLS_-FP_N_ and P_NLS_-FP_C_, respectively.

For the 12 copies of the MBS-PBS hybrid stem loop sequence (HSL12), the Pcr4-12X-MBS-PBS [15] DNA was digested with BamHI and SpeI, followed by the Klenow fill-in reaction. The 1402-bp HSL12 fragment was gel-eluted and ligated with the binary vector pCAMBIA1390 containing the *35S-FT* or *35S-RFP* to form the p1390-35S-*FT*_*HSL12*_ or p1390-35S-*RFP*_*HSL12*_ construct, respectively.

### Agro-infiltration of *N. benthamiana*

*Agrobacterium tumefaciens* strain AGL1 carrying individual constructs was cultured in 20 mL LB broth (10 g/L tryptone, 5 g/L yeast extract, 10 g/L NaCl) containing 50 μg mL^−1^ kanamycin, 10 mM MES, pH 5.7 and 20 μM acetosyringone at 28 °C with 220 rpm shaking for 2 days. Bacteria were pelleted by centrifugation and resuspended in infiltration medium (10 mM MgCl2, 10 mM MES, pH 5.7, and 200 μM acetosyringone) to OD600 = 1.0. The bacterial solution was placed at room temperature for at least 1 h. For agro-infiltration, the bacteria solution was infiltrated into the abaxial side of the 4^th^ or 5^th^ leaves of 3-week-old *N. benthamiana* by using a needle-removed 5-mL syringe. For co-infiltration with 2 or 3 different constructs, different bacterial solution or infiltration media were mixed at a 1:1 volume ratio and infiltrated into leaves. The infiltrated plants were cultured in growth chambers for 3 days, then infiltrated leaves were excised for confocal laser scanning microscopy.

### Confocal laser scanning microscopy

GFP fluorescence was observed by confocal laser scanning microscopy (LSM880, Carl Zeiss) with the argon laser set to 488/500–530 for excitation/emission.

## Data Availability

All data and materials are available upon request to TSY (tienshin@gate.sinica.edu.tw').

## Abbreviations

FP: Fluorescent protein
MCP: MS2 bacteriophage coat protein
PCP: PP7 bacteriophage coat protein
MBS: MCP binding sequences
PBS: PCP binding sequences
NLS: Nuclear localization sequence
HSL: MBS-PBS hybrid stem loop sequences
